# Nimesulide, a COX-2 inhibitor, sensitizes pancreatic cancer cells to TRAIL-induced apoptosis by promoting DR5 clustering †

**DOI:** 10.1080/15384047.2023.2176692

**Published:** 2023-02-12

**Authors:** Nagamani Vunnam, Malaney C. Young, Elly E. Liao, Chih Hung Lo, Evan Huber, MaryJane Been, David D. Thomas, Jonathan N. Sachs

**Affiliations:** a Department of Biomedical Engineering, University of Minnesota, Minneapolis, MN, USA; b Department of Biochemistry, Molecular Biology, and Biophysics, University of Minnesota, Minneapolis, MN, USA

**Keywords:** Nimesulide, COX-2 inhibitors, pancreatic cancer cells, tumor necrosis factor receptors, death receptor 5, TNF-related apoptosis inducing ligand

## Abstract

Nimesulide is a nonsteroidal anti-inflammatory drug and a COX-2 inhibitor with antitumor and antiproliferative activities that induces apoptosis in oral, esophagus, breast, and pancreatic cancer cells. Despite being removed from the market due to hepatotoxicity, nimesulide is still an important research tool being used to develop new anticancer drugs. Multiple studies have been done to modify the nimesulide skeleton to develop more potent anticancer agents and related compounds are promising scaffolds for future development. As such, establishing a mechanism of action for nimesulide remains an important part of realizing its potential. Here, we show that nimesulide enhances TRAIL-induced apoptosis in resistant pancreatic cancer cells by promoting clustering of DR5 in the plasma membrane. In this way, nimesulide acts like a related compound, DuP-697, which sensitizes TRAIL-resistant colon cancer cells in a similar manner. Our approach applies a time-resolved FRET-based biosensor that monitors DR5 clustering and conformational states in the plasma membrane. We show that this tool can be used for future high-throughput screens to identify novel, nontoxic small molecule scaffolds to overcome TRAIL resistance in cancer cells.

## Introduction

Tumor necrosis factor-related apoptosis-inducing ligand (TRAIL) selectively induces apoptosis in cancer cells via engagement of its cognate receptor, death receptor 5 (DR5).^1^ However, a significant number of cancer cells are resistant to TRAIL; particularly some highly malignant cancers such as melanoma and pancreatic cancer.^2,3^ Several novel drugs targeting TRAIL receptors are currently in clinical trials,^4^ though TRAIL resistance limits their effectiveness. The current mechanistic understanding of TRAIL resistance remains muddied, with several overlapping factors likely conspiring to confer a resistant phenotype. These factors include, among others: altered surface expression of death receptors;^5,6^ the inability of the receptors to initiate death signal due to mutations;^7^ defects in adaptor proteins which are essential for assembly of the death-inducing signaling complex;^8,9^ and upregulation of apoptosis inhibiting proteins.^10,11^ Regardless of its origins, there have been considerable efforts to develop therapeutic strategies that circumvent or overcome TRAIL-resistance.^12^

One surprising advance came in the study of cyclooxygenase-2 (COX-2) inhibitors. COX-2 is a central enzyme in the production of proinflammatory prostaglandin from arachidonic acid, and is known to inhibit apoptosis and immune surveillance, stimulate angiogenesis, promote cancer invasion and metastasis.^13–15^ Accordingly, COX-2 is considered a therapeutic target for prevention of several cancers including hepatocellular, colorectal, and pancreatic cancers.^16–19^ Previous studies have shown that COX-2 inhibitors sensitize cancer cells to death receptor ligands including Fas, tumor necrosis factor and TRAIL.^20,21^ For example, DuP-697, a selective COX-2 inhibitor, sensitizes TRAIL resistant colon carcinoma cell to apoptosis by promoting the clustering of DR5 in the plasma membrane.^20^ More recently, nimesulide, another COX-2 inhibitor and the subject of this study, has been associated with antitumor and an antiproliferative effect.^22,23^ Nimesulide is a nonsteroidal anti-inflammatory drug that induces apoptosis in breast cancer, oral and esophagus carcinoma cells, inhibits lung cancer cell proliferation and suppresses gastric carcinogenesis.^24–28^ Recent studies also showed that nimesulide inhibits proliferation and induces apoptosis of pancreatic cancer cells by upregulating the tumor suppressor gene PTEN (phosphatase and tensin homolog deleted on chromosome 10).^29–31^ Whether nimesulide also has the effect of sensitizing resistant pancreatic cancer cells to TRAIL is unknown.

Here, we investigated the effects of nimesulide on TRAIL sensitivity in human lymphoma Jurkat cells and two TRAIL-resistant pancreatic cancer cell lines, AsPC1 and Panc1, all of which express DR5 on their surface. Cell-based functional assays confirm that AsPC1 and Panc1 cancer cells are resistant to TRAIL-induced apoptosis, and show that co-treatment of TRAIL with nimesulide confers dose-dependent TRAIL sensitivity (as measured by caspase-8 activation). Using fluorescence microscopy, we illustrate that nimesulide’s enhancement of TRAIL-induced apoptosis, like DuP-697, correlates with clustering of DR5 in the plasma membrane. These clusters are thought to be essential to DR5 activation, and they reflect receptor association with lipid rafts and concomitant backbone conformational changes.^20,32^ Using nimesulide and a second positive control, bioymifi, we then show that small-molecule induced clustering of DR5 can be monitored through a FRET-based DR5 biosensor we previously developed.^33^ Building on that technology, we provide a first proof-of-principle that this FRET platform can be extended for future high-throughput screening campaigns aimed at identifying novel scaffolds for enhancing TRAIL-sensitivity in resistant cells.

## Materials and methods

### Cell cultures and reagents

HEK293 cells were cultured in phenol red-free DMEM (Gibco) supplemented with 2 mM L-Glutamine (Invitrogen). Jurkat and AsPC1 cell line were cultured in RPMI 1640 with HEPES, sodium pyruvate, and L-glutamine (ATCC). Panc1 cells were cultured in Dulbecco’s Modified Eagle’s Medium with 4 mM L-glutamine, 4500 mg/L glucose, 1 mM sodium pyruvate, and 1500 mg/L sodium bicarbonate (ATCC). All media was supplemented with heat-inactivated 10% fetal bovine serum (Gibco), 100 U/mL penicillin and 100 mg/mL streptomycin (HyClone). Cell cultures were maintained in an incubator with 5% CO_2_ (Forma Series II Water Jacket CO_2_ Incubator: Thermo Fisher Scientific) at 37°C.

### Molecular cloning

TagRFP and EGFP plasmids were prepared as described previously.^34^ Genes encoding DR5ΔCD (1–240) were inserted at the N-terminus of the TagRFP and EGFP vectors using standard cloning techniques, as described previously.^35^ To prevent the dimerization and aggregation of EGFP, alanine 206 was mutated to lysine (A206K).^36,37^

### Detection of endogenous expression DR5 and DR4 on Jurkat and pancreatic cells

To examine the surface expression of DR5, cancer cells were washed 3x with cold PBS and incubated with allophycocyanin-conjugated anti-DR5 antibody for 1–2 hours on ice. Cells were washed three times with cold PBS and surface expression of DR5 was determined using flow cytometry. To test the effect of nimesulide on surface expression of DR5, cancer cells were seeded in a 10 ml flask and incubated for 24 hours at 37°C. Cells were treated with nimesulide (50 or 100 μM) and DMSO only controls and incubated for 24 hours at 37°C. Cells were washed three times with cold PBS and incubated with anti-DR5 antibody for 1–2 hours on ice. Cells were washed three times with cold PBS and surface expression of DR5 was determined using flow cytometry.

### Western blot

Expression of DR5 and DR4 in cancer cells was also determined using a Western blot. Cancer cells were seeded in a 10 ml flask and incubated for 24 hours at 37°C. Cells were treated with (50 μM) and without nimesulide and incubated for 24 hours at 37°C. After incubation, cells were spun down and washed three times with cold PBS and lysed with native lysis buffer. Total protein concentration of lysates was determined by BCA assay, and equal amounts of total protein (20–80 μg) were mixed with 4x Bio-rad sample buffer and boiled for 3–5 min and resolved using 4–20% Tris–glycine SDS-PAGE gels (Bio-rad). Proteins were transferred to a PVDF membrane (EMD Millipore) and probed using antibodies against DR5, DR4, and β-Actin (Cell signaling technology and Biolegend). SHSY5Y cell lysate was used as a positive control for DR4.

### Caspase-8 activity assay

The caspase-8 assay was used to assess the effects of TRAIL only, nimesulide only, and nimesulide+TRAIL on caspase-8 activity, and nimesulide effective concentration. Cancer cells were seeded in 96-well white opaque plates (Greiner Bio-One) at 7,500 cells/well and incubated for 24 hours at 37°C. Cells were incubated for 24 hours at 37°C with TRAIL alone (0.000001–10 μg/mL) or nimesulide alone (0.001–200 μM) or with nimesulide (50 μM) + TRAIL (0.000001–10 μg/mL) or DMSO only. An equal volume of Caspase-Glo 8 reagent (Promega) was added to each well, and the luminescence was measured after 45 minutes using a Cytation 3 Cell Imaging Multi-Mode Reader luminometer (BioTek). To determine the effective concentration (EC50) of nimesulide, Jurkat cells were seeded in 96-well white opaque plates at 7,500 cells/well and incubated for 24 h at 37°C. Cells were treated for 2 hours with nimesulide (0.001–200 μM) before being treated for 24 hours with TRAIL (0.01–0.1 μg/mL) at 37°C. An equal volume of Caspase-Glo 8 reagent (Promega) for each sample was added to each well, and the luminescence was measured after 45 min.

### TRAIL binding assay

Jurkat cells were washed with PBS, and then cells (450,000/condition) were incubated with flag-tagged TRAIL (0.1 μg/mL) ± the nimesulide (EC_50_ concentration) for 2 hours on ice until equilibrated. After incubation, cells were washed two times with PBS to remove unbound TRAIL. Cells were labeled with rabbit anti-flag antibody, followed by AF647-conjugated anti-rabbit secondary antibody. TRAIL binding was measured using BD Accuri C6 flow cytometer.

### DR5 clustering assay

To examine the effect of Nimesulide on DR5 clustering, HEK293 cells were transfected with the DR5ΔCD-RFP (7.5 μg) in a 10 cm plate using Lipofectamine 3000 (Invitrogen). After 24 hours of transfection, cells were lifted with TrypLE, resuspended in phenol red–free DMEM (Gibco) and centrifuged for 5 minutes at 200 g. Transfected cells were dispensed in a 24-well glass black clear bottom plate. The next day, transfected cells were treated separately with DMSO, bioymifi, TRAIL, nimesulide, or nimesulide+TRAIL and incubated overnight. Cells were fixed with 4% paraformaldehyde for 10–15 minutes and washed with PBS and imaged using a ImageXpress Pico imaging system. Panc1 cells, which have endogenous expression of DR5, were dispensed in a 24-well glass black clear bottom plate and treated separately with DMSO, bioymifi, TRAIL, nimesulide or nimesulide+TRAIL and incubated overnight. Next day cells were fixed with 4% paraformaldehyde for 10–15 minutes and washed with PBS, then labeled with APC-conjugated anti-DR5 antibody. Cells were mounted with antifade mounting medium with DAPI. Fluorescence images of cells were taken using ImageXpress Pico imaging system.

### Pilot screening with LOPAC library

The LOPAC library, containing 1280 compounds, was purchased from Sigma-Aldrich. 384-well flat bottom polypropylene black plates were selected as the assay plates for their low autofluorescence and low inter-well cross talk (Greiner Bio-One). LOPAC compounds were formatted across four 384-well plates at 50 nL (10 μM final concentration/well) from 96-well mother plates using an Echo liquid dispenser. Plates were sealed and stored at −20°C until use. For screening, HEK293 cells were transfected using Lipofectamine 3000 with DR5ΔCD-GFP and DR5ΔCD-GFP:DR5ΔCD-RFP (1:6 ratio) for a total of 15.75 µg in 10 cm plates for 24 hours and transfection was confirmed using EVOS fluorescence microscopy. On the day of screening, drug plates were equilibrated at room temperature. The cells were harvested from the 10 cm plates by incubating for 2–4 minutes with TrypLE (Invitrogen), washed three times with PBS, filtered using 70 μm cell strainers (BD Falcon), and resuspended in PBS at a concentration of 1 million cells/mL. Next, cells were dispensed (50 μL/well) into the 384-well LOPAC drug plates (Greiner) by a Multidrop Combi Reagent Dispenser (Thermo Fisher Scientific) and allowed to incubate for 2 hours at room temperature before readings were acquired. Donor lifetime in the presence and absence of acceptor was measured by using a fluorescence lifetime plate reader (Fluorescence Innovations, Inc., Minneapolis, MN). GFP fluorescence was excited with a 473-nm microchip laser, and emission was filtered with 488-nm long-pass and 517/20-nm band-pass filters. Time-resolved fluorescence waveforms for each well were fitted to single-exponential decays using least-squares minimization global analysis software (Fluorescence Innovations, Inc.) to give donor lifetime (τ_D_) and donor–acceptor lifetime (τ_DA_). FRET efficiency (E) was then calculated based on Eq. (1)

(1) $$E=1-\left(\frac{\tau_{DA}}{\tau_{D}}\right)\;$$

### MTT assay

The 3-(4,5-dimethylthiazol-2-yl)-2,5-diphenyltetrazolium bromide (MTT) assay was used to measure apoptosis and cell proliferation. For apoptosis measurements, pancreatic cancer cells were seeded in 96-well plates at a density of 7500 cells/well and incubated for 24 hours at 37°C and 5% CO2. After incubation, cells were treated with increasing concentrations of TRAIL (0.000001–10 μg/ml) + DMSO or nimesulide (50 μM) + TRAIL (0.000001–10 μg/ml), followed by 24 hours of incubation at 37°C. For cell proliferation, cancer cells were seeded in 96-well plates at a density of 7500 cells/well and incubated for 24 h at 37°C and 5% CO2. After incubation, cells were treated with increasing concentrations of nimesulide (0.001–200 μM), followed by 24 hours of incubation at 37°C. Cell viability and proliferation were assessed with a Cytation 3 Cell Imaging Multi-Mode Reader luminometer (BioTek).

### Overexpression of FADD in HEK293 cells

Human-FADD plasmid was purchased from Origene technologies. HEK293 cells were transfected with the FADD plasmid (15 μg) in a 10 cm plate using Lipofectamine 3000 (Invitrogen). After 48 hours of transfection, cells were lifted with TrypLE, resuspended in phenol red–free DMEM (Gibco) and centrifuged for 5 minutes at 200 g. Transfected cells were washed three times with cold PBS and lysed with native lysis buffer (Abcam). Overexpression of FADD was confirmed by western blot with anti-FADD antibody (Cell Signaling).

### Apoptosis induced by overexpression of FADD

To determine the effect of nimesulide on apoptosis induced by overexpression of FADD, HEK293 cells were transfected with the FADD plasmid (15 μg) in a 10 cm plate using Lipofectamine 3000 (Invitrogen). After 24 hours of transfection, cells were lifted with TrypLE and resuspended in phenol red–free DMEM (Gibco). Next, transfected cells were plated in 96-well white opaque plates (7,500 cells/well) and incubated for 24 hours at 37°C. Cells were then treated with nimesulide (0.001–200 μM) and incubated for 24 hours at 37°C. An equal volume of Caspase-Glo 8 reagent (Promega) to sample was added to each well, and the luminescence was measured after 45 min using a Cytation 3 Cell Imaging Multi-Mode Reader luminometer (BioTek).

### DR5 knockdown using DR5 siRNA

For DR5 knockdown experiments, DR5 siRNA Gene Silencer was purchased from Santa Cruz Biotechnology (sc-40237, Santa Cruz Biotechnology). Cells were transfected with DR5 siRNA (25 nM) using lipofectamine 3000 (Invitrogen) according to the manufacturer’s recommendations. Transfected cells were incubated for 48 hours before further analysis. DR5 expression was determined using an anti-DR5 antibody and analyzed by flow cytometry.

## Results

### Varied TRAIL sensitivity of cancer cell lines

**Figure 1.**
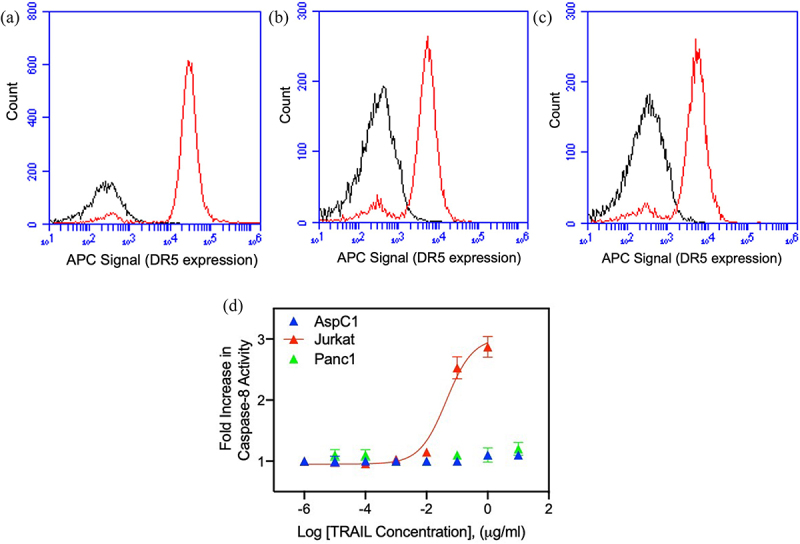
Surface expression of DR5 and TRAIL sensitivity of Jurkat and pancreatic cancer cells. FACS data demonstrate surface expression of DR5 on Jurkat (a), AsPC1(b) and Panc1 (c) cells. Cells were incubated with the APC-conjugated anti-DR5 antibody and analyzed by flow cytometry. The red line indicates cells labeled with anti-DR5 antibody and black line for unlabeled cells. (d) TRAIL sensitivity of AsPC1, Panc1 and Jurkat cancer cells. Caspase-8 activity was measured in Jurkat, and pancreatic cancer cells treated with increasing concentrations of TRAIL (0.000001–10 μg/ml). Data are presented as mean ± standard deviation (N = 3).

It is well documented that TRAIL induces apoptosis in various types of cancer cells via engaging its cognate receptor DR5.^38^ However, not all cancer cells are sensitive to TRAIL, which could be due to the lack of DR5 expression. So, before testing the effect of nimesulide on TRAIL-induced apoptosis, we first examined the surface expression of DR5 in human lymphoma Jurkat cells and two pancreatic cancer cells, AsPC1 and Panc1. Flow cytometry data showed that all three cell lines were positive for DR5 expression (Figure 1a-c). However, DR5 expression was slightly higher in Jurkat cells (Figure 1a) when compared with AsPC1 (Figure 1b) and Panc1 (Figure 1c).

Next, we tested the sensitivity of Jurkat and pancreatic cancer cells to TRAIL using a caspase-8 activation assay. Studies have shown that upon TRAIL binding DR5 triggers apoptosis by recruiting the apoptosis initiator caspase-8 through the adaptor FADD.^39^ Caspase-8 activity was increased in Jurkat cells in the presence of TRAIL in a dose-dependent manner, whereas the AsPC1 and Panc1 showed very minimal sensitivity to TRAIL (Figure 1d). These results confirm that the AsPC1 and Panc1 cells are resistant to TRAIL despite their intact expression of DR5. As previously mentioned, TRAIL resistance can be due to a wide variety of mechanisms, including defects in death inducing signaling complex proteins like FADD and caspase-8.^8–10^

### Nimesulide increases TRAIL-induced caspase-8 activity in Jurkat cells

**Figure 2.**
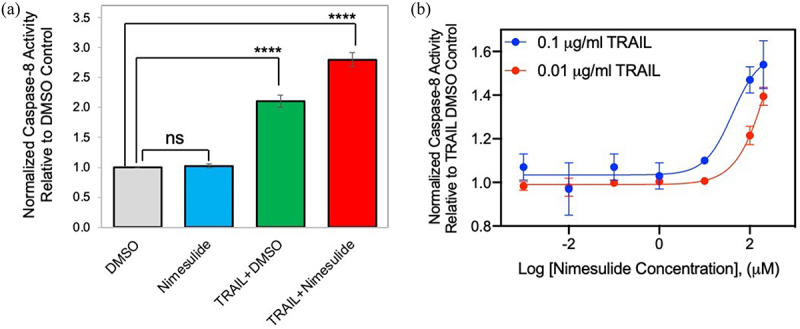
Effect of nimesulide on TRAIL-induced caspase-8 activity. (a) Caspase-8 activity was measured in Jurkat cells treated with nimesulide-only (50 μM), TRAIL (0.1 μg/ml) +DMSO or TRAIL+nimesulide (50 μM) or DMSO only. (b) Determination of EC_50_ of nimesulide by caspase-8 assay. Jurkat cells were incubated with increasing concentrations of nimesulide (0.001–200 uM) and TRAIL (0.01–0.1 μg/ml). Data are presented as mean ± standard deviation (N = 3). ****P < .0001 compared to control by two-tailed unpaired t test.

To test the biological effect of nimesulide on TRAIL-induced apoptosis, the caspase-8 activation assay was performed with Jurkat cells. These results show that nimesulide increases the TRAIL-induced caspase-8 activity by 1.4-fold compared to DMSO+TRAIL treated cells (Figure 2a). However, cell treated with nimesulide-only did not affect the caspase-8 activity compared to DMSO control (Figure 2a). These results suggest that nimesulide and TRAIL work synergistically to increase caspase-8 activity. Next, we determined the effective concentration (EC_50_) of nimesulide in the presence of TRAIL (0.01 and 0.1 μg/ml) using the caspase-8 assay. As shown in Figure 2b, nimesulide increased caspase-8 activity in a dose-dependent manner, with an EC_50_ of 57 μM at 0.1 μg/ml TRAIL and 915 μM at 0.01 μg/ml TRAIL. Taken together, nimesulide enhances TRAIL-induced caspase-8 activity in Jurkat cells.

### Nimesulide sensitizes pancreatic cancer cells to TRAIL-induced caspase-8 activity

**Figure 3.**
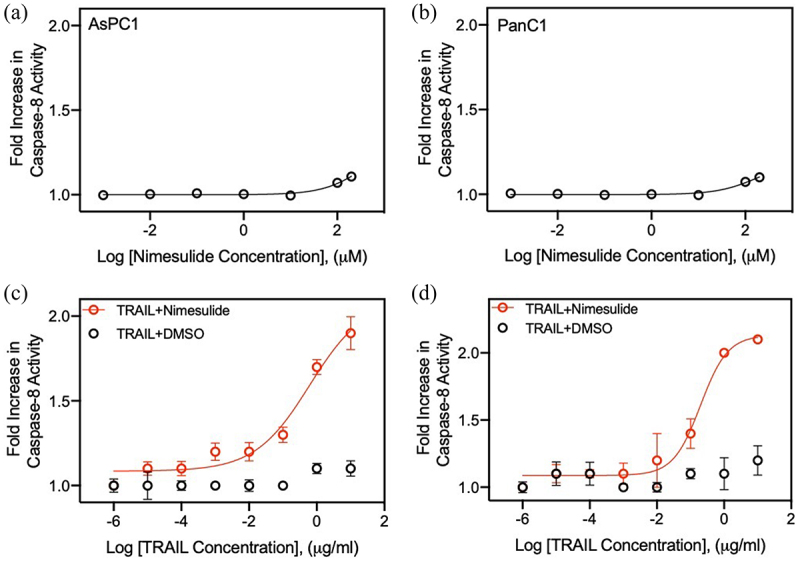
Effect of nimesulide on caspase-8 activity in AsPC1 and Panc1 cancer cells. Caspase-8 activity was measured in AsPC1 (a) and Panc1 (b) cancer cells treated with increasing concentrations of nimesulide-only (0.001–200 μM). (c) AspC1 cells treated with increasing concentrations of TRAIL (0.000001–10 μg/ml) +DMSO or TRAIL+nimesulide (50 μM). (d) PanC1 cells treated with increasing concentrations of TRAIL (0.000001–10 μg/ml) +DMSO or TRAIL+nimesulide (50 μM). Data are presented as mean ± standard deviation (N = 3).

To determine the promise of nimesulide for clinical translation, we tested its ability to sensitize AsPC1 and Panc1 cells to TRAIL-induced caspase-8 activity. We first investigated whether nimesulide alone activates caspase-8 in these cell lines. As shown in Figure 3, nimesulide-only treatment resulted in a 10% increase in caspase-8 activity at a high dose (200 M) in AsPC1 (Figure 3a) and Panc1 (Figure 3b). However, co-treatment of TRAIL with nimesulide (50 μM) increased caspase-8 activity by 1.7-fold over DMSO in AsPC1 (Figure 3c) and by 2-fold over DMSO in Panc1 cells (Figure 3d). These results confirm that nimesulide increases TRAIL-induced caspase-8 activity in pancreatic cancer cells.

### Nimesulide increases TRAIL-induced apoptosis in pancreatic cancer cells

**Figure 4.**
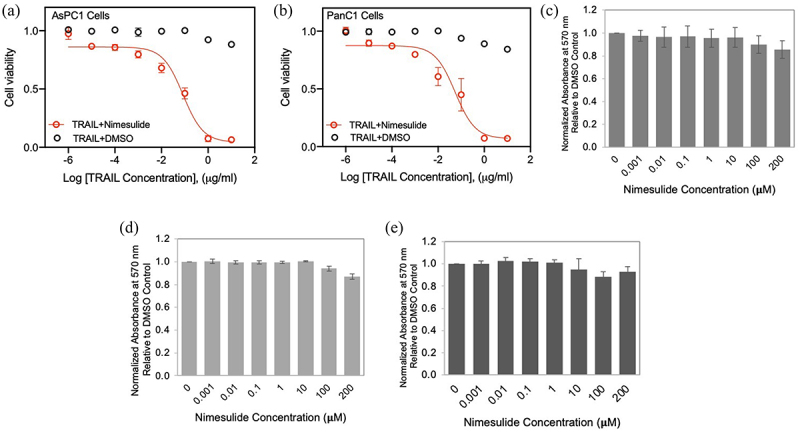
Effect of nimesulide on TRAIL-induced apoptosis in pancreatic cancer cells and proliferation of all three cancer cells. Apoptosis was measured using MTT assay in AsPC1 (a) and Panc1 (b). Cancer cells were treated with increasing concentrations of TRAIL (0.000001–10 μg/ml) +DMSO or TRAIL+nimesulide (50 μM). Data are presented as mean ± standard deviation (N = 3). AsPC1 (c), Panc1 (d) and Jurkat (e) cells were treated separately with DMSO or nimesulide (0.001–200 μM) and incubated for 24 hours and cell proliferation was measured using MTT assay. Data are mean ± SD (N = 3).

Previous studies have reported that nimesulide promotes apoptosis in several different cancer cells.^25,26^ So, we measured the effect of nimesulide on TRAIL-induced apoptosis in AsPC1 and Panc1 cells using MTT assay. While TRAIL-only treatment showed a 12% increase in cell death at a high concentration (10 μg/ml), co-treatment of TRAIL with nimesulide (50 μM) increased apoptosis by >90% in both AsPC1 (Figure 4a) and Panc1 cells (Figure 4b). These results clearly confirm that nimesulide sensitizes pancreatic cancer cells to TRAIL-induced apoptosis.

### Effect of nimesulide on cell proliferation

It has been reported that nimesulide inhibits cell proliferation and suppresses carcinogenesis in several different cancer cells.^29^ So, we sought to investigate the effect of nimesulide on Jurkat and pancreatic cell proliferation using the MTT assay. MTT assay was developed as a non-radioactive alternative to tritiated thymidine incorporation into DNA for measuring cell proliferation.^40^ These studies showed that low doses of nimesulide did not affect the proliferation of all three cancer cells (Figure 4c-e). However, 200 μM treatment decreased cell proliferation by 20%. In spite of this anti-proliferative effect at a high dose, we observed an increase in caspase-8 activity and apoptosis (Figure 3 and Figure 4a-b). These findings demonstrate that cell proliferation is not the source of nimesulide proapoptotic activity.

### Effect of nimesulide on TRAIL binding

**Figure 5.**
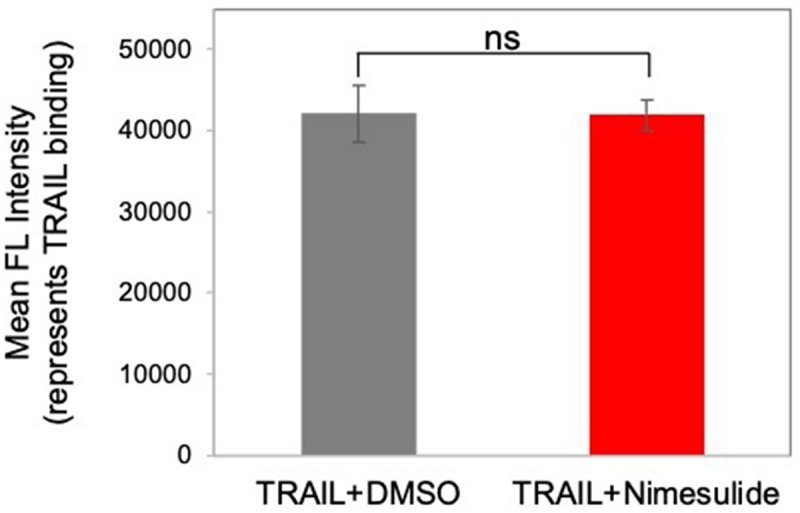
Effect of nimesulide on TRAIL binding. Jurkat cells were incubated with TRAIL (0.1 μg/ml) +DMSO or TRAIL+nimesulide (effective dose concentration). TRAIL binding was detected with rabbit anti-Flag antibody, followed by AF647-conjugated anti-rabbit secondary antibody, as measured by flow cytometry. Data are presented as mean ± standard deviation (N = 3).

We tested whether antitumor effect of nimesulide is coming from an increase in TRAIL binding. So, we examined the effect of nimesulide on TRAIL binding using flow cytometry. This assay was performed at the effective dose of the nimesulide. No significant difference was observed in the binding of TRAIL to Jurkat cells in the presence versus absence of nimesulide (Figure 5). These results confirm that nimesulide is not altering TRAIL binding.

### Nimesulide enhances TRAIL-induced capase-8 activity in pancreatic cancer cells by promoting DR5 clustering

**Figure 6.**
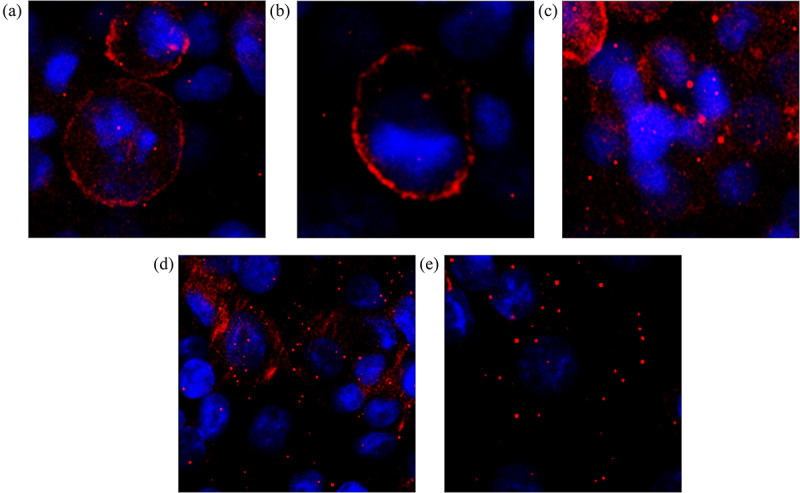
Effect of nimesulide on DR5 clustering. Panc1 cells were treated separately with DMSO (a), TRAIL+DMSO (b), bioymifi (c), nimesulide (d) and nimesulide+TRAIL (e). The next day cells were fixed with 4% paraformaldehyde and then labeled with APC-conjugated anti-DR5 antibody. Cells were mounted with antifade mounting medium with DAPI. Fluorescence images of cells were taken using ImageXpress Pico imaging system.

Previously studies reported that death receptor clustering at the cell membrane is required for the initiation of death signaling and is an important factor for TRAIL-induced apoptosis.^41–44^ A recent study showed that DuP-697, a selective COX-2 inhibitor, sensitizes human colon carcinoma cells to TRAIL-mediated apoptosis via clustering of DR5 in cholesterol enriched and ceramide-enriched caveolae.^20^ Also, a small molecule bioymifi increases apoptosis in several different cancer cells by promoting ligand-independent DR5 clustering.^45^ So, to determine the mode of action of nimesulide, we first tested its effect on DR5 clustering in Panc1 cells using the ImageXpress Pico imaging system. Fluorescence microscopy data showed that DR5 clustering was significantly increased in cells treated with bioymifi (positive control, Figure 6c) and nimesulide (Figure 6d) when compared with DMSO control (Figure 6a). Interestingly, co-treatment of TRAIL with nimesulide further enhanced DR5 clustering (Figure 6e and Supplementary Figure 2B). However, DR5 clustering was not increased in Panc1 cells treated with TRAIL alone (Figure 6b and Supplementary Figure 2a) in comparison with control (Figure 6a). These results suggest that nimesulide increases the ligand-independent and -dependent clustering of DR5.

**Figure 7.**
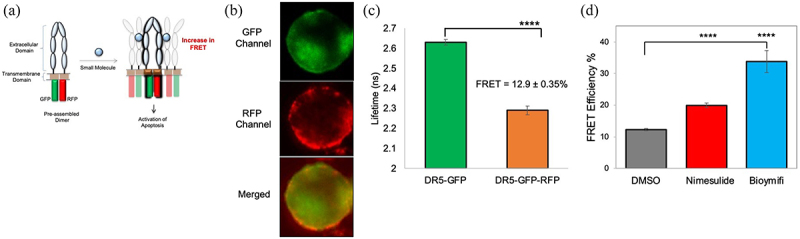
Effect of nimesulide on DR5-DR5 interactions. (a) Schematic of DR5ΔCD-FRET pair biosensor. Pre-ligand assembled DR5 dimers show FRET, which will be increased if small molecules are promoting DR5 clustering. (b) Fluorescence microscopy images showing the expression of DR5ΔCD-GFP and DR5ΔCD-RFP in cells transfected with DR5ΔCD-FRET pair (c) Fluorescence lifetime measurements with HEK293 cells transiently expressing DR5ΔCD-GFP only and DR5ΔCD-FRET pair. (d) FRET efficiency increased with nimesulide and bioymifi treatment compared to DMSO control. Data are mean ± SD (N = 3). ****P < .0001 compared to DMSO control by two-tailed unpaired t test.

To further corroborate the effect of nimesulide on receptor–receptor interactions, we tested its effect on pre-ligand receptor–receptor interactions using TR-FRET method. We have previously used this method to monitor DR5-DR5 interactions.^33^ TR-FRET experiments were performed in HEK293 cells transiently expressing DR5 without a cytoplasmic domain (DR5ΔCD) attached to GFP and coexpressing DR5ΔCD fused to GFP and RFP just downstream of the transmembrane domain of the receptors (Figure 7a). Fluorescence images of HEK293 cells co-expressing DR5ΔCD-GFP and DR5ΔCD-RFP (DR5ΔCD-FRET pair) showed an overlap of the GFP and RFP, indicating the colocalization of the receptors (Figure 7b). Lifetime measurements showed a substantial decrease in the DR5ΔCD-GFP (donor) fluorescence lifetime in the presence of the DR5ΔCD-RFP (acceptor) compared with the donor only, which confirms the efficient energy transfer between the FRET pairs (Figure 7c). This data recapitulates our previous finding that DR5ΔCD receptors are capable of homophilic interactions in the absence of cytoplasmic death domain and ligand.^46–48^ FRET efficiency was significantly increased in cells treated with bioymifi (positive control) or nimesulide when compared with DMSO control (Figure 7d).

We next investigated if increase in FRET efficiency is due to DR5 clustering. For this study, we used HEK293 cells transiently expressing DR5ΔCD-RFP. Fluorescence microscopy data showed that DR5 clustering was significantly increased in cells treated with bioymifi (positive control, Supplementary Figure 1C) and nimesulide (Supplementary Figure 1D) when compared with DMSO control (Supplementary Figure 1A). Unlike Panc1 cells, DR5 clustering was increased in HEK293 cells treated with TRAIL compared to DMSO control (Supplementary Figure 1A-B). In HEK293 cells also, co-treatment of TRAIL with nimesulide further increased DR5 clustering (Supplementary Figure 1E). Taken together, these data confirm that nimesulide sensitizes pancreatic cancer cells to TRAIL-induced apoptosis by promoting DR5 clustering.

**Figure 8.**
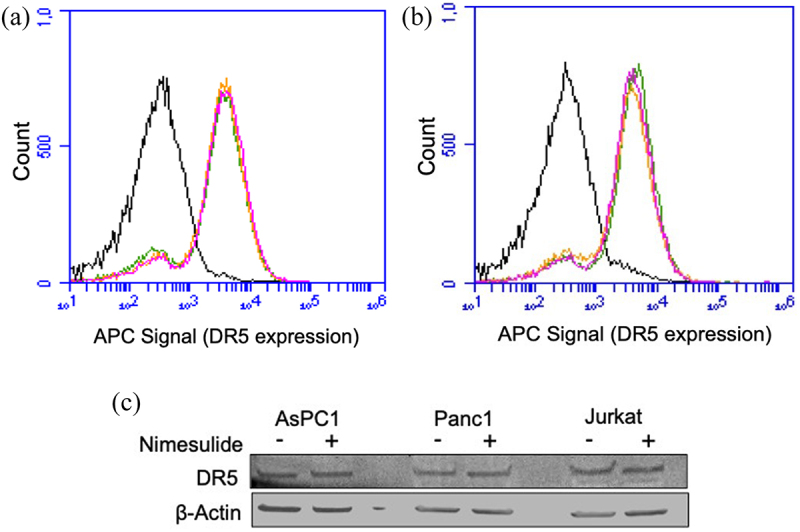
Effect of nimesulide on DR5 expression. FACS data demonstrate surface expression of DR5 on AsPC1 (a) or Panc1 (b) cells. Cells were treated separately with DMSO or nimesulide and incubated for 24 hours. After incubation cells were washed with PBS and incubated with APC-conjugated anti-DR5 antibody and analyzed by flow cytometry. The black line indicates for unlabeled cells, green for cells treated with DMSO and Orange (50 μM) and magenta (100 μM) for cells treated with nimesulide. (c) Cancer cells were treated with or without nimesulide (50 μM) and incubated for 24 hours. After incubation cells were washed with PBS and lysed with native lysis buffer. Equal amounts of total protein were loaded and resolved by 4–20% Tris-glycine SDS−PAGE gels and immunoblotted with antibodies against DR5 and β-Actin.

We next sought to examine if increase in DR5 clustering is due to upregulation of DR5 expression. Studies have shown that small molecules like Chloroquine and 3,3’-diindolylmethane enhances TRAIL-mediated apoptosis through upregulation of DR5.^49,50^ Surface expression of DR5 was examined in the presence and absence of nimesulide using flow cytometry (Figure 8a-b) and western blot (Figure 8c and supplementary Figure 3A). As shown in Figure 8, nimesulide treatment did not affect the expression of DR5 in cancer cell lines. These results confirm that the expression level of DR5 was not involved in sensitization of these cancer cells to TRAIL.

### Effect of nimesulide on surface expression of DR4

To further verify the specificity of nimesulide, we tested its effect on expression of death receptor 4 (DR4), which is a homologous member of the TNF receptor superfamily (51% identical and 68% like DR5) that binds to TRAIL and triggers apoptosis.^51,52^ We tested the expression of DR4 in all three cancer cell lines in the presence and absence of nimesulide using a western blot. It has been reported that the neuroblastoma cell-line SHSY5Y expresses both DR5 and DR4,^53^ so we have used the lysate from SHSY5Y cells as a positive control. No significant expression of DR4 was observed in the presence or absence of nimesulide (Supplementary Figure 3B-C) in all three cancer cell lines. Some studies have previously shown that these cancer cell lines have very low or no DR4 expression.^54,55^ Furthermore, it has been reported that TRAIL-induced apoptosis is preferentially mediated via DR5 in AsPC-1 and Panc1.^54,55^ These results suggest that DR4 is not contributing to TRAIL-induced apoptosis, and nimesulide does not affect the DR4 expression levels in all these cell lines.

### Effect of nimesulide FADD overexpression-induced apoptosis

**Figure 9.**
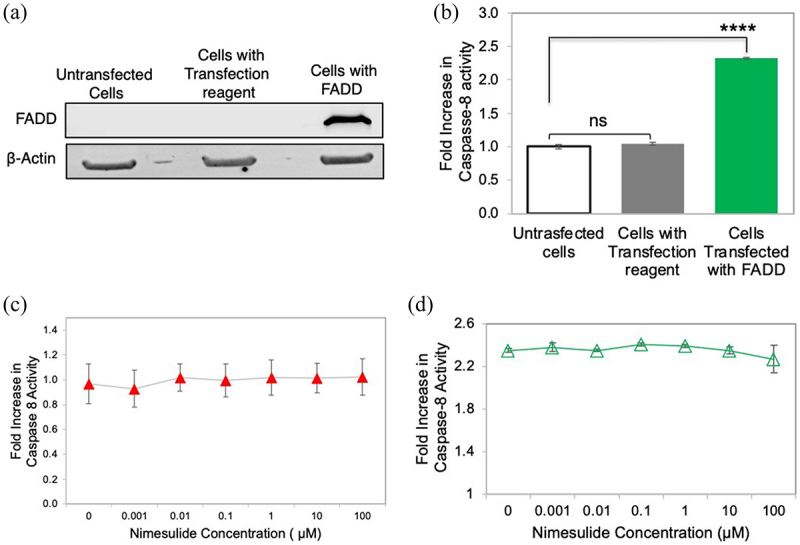
Effect of nimesulide on FADD overexpression-induced apoptosis in HEK293 cells. (a) Western blot analysis of HEK293 cell lysates of untransfected and FADD transfected cells (b) Overexpression of FADD increases caspase-8 activity in HEK293 cells. (c) Effect of nimesulide on endogenous caspase-8 activity in HEK293 cells. (d) Effect of nimesulide on FADD-induced caspase-8 activity in HEK293 cells overexpressing FADD. Data are presented as mean ± standard deviation (N = 3). ****P < .0001 compared to control by two-tailed unpaired t test.

To rule out nonspecific downstream signaling effects of nimesulide, we tested their effects on FADD overexpression-induced apoptosis. DR5-independent apoptosis pathways can be interrogated in HEK293 cells due to their negligible expression of DR5.^56,57^ It has been previously shown that overexpressing FADD in HEK293 cells induces apoptosis-independent of the TRAIL-DR5 pathway.^58–60^ Thus, any effect on FADD-induced apoptosis in the presence of the small molecules suggests that they are acting through a mechanism that is not DR5-dependent. To test the effects of DR5 hit compounds on FADD overexpression-induced apoptosis, we first confirmed that the FADD plasmid could induce overexpression of FADD in HEK293 cells, which was determined using western blot (Figure 9a and Supplementary Figure 4). Transfected cells showed a significant band at 23 kDa corresponding to FADD compared to untransfected cells (Supplementary Figure 4). Next, we tested the effect of FADD overexpression on caspase-8 activity. Caspase-8 activity was significantly increased (2.5-fold) in cells transfected with FADD when compared with untrasfected HEK293 cells (Figure 9b)

In order to establish that the mode of action of nimesulide occurs through DR5 and not an independent caspase-8 pathway, we tested the effects of the nimesulide on basal caspase-8 activity in HEK293 cells, which have very low levels of endogenous DR5.^56,57^ Nimesulide had no effect on basal caspase-8 activity in HEK293s (Figure 9c), which suggests that nimesulide is not increasing caspase-8 activity in a DR5-independent manner. We then tested the effects of nimesulide on FADD-induced apoptosis, to determine whether the changes in caspase-8 activity are due to a nonspecific interaction with FADD. Nimesulide had little to no effect on FADD-induced apoptosis, even at relatively high concentrations (Figure 9d). These results confirm that the mode of action of nimesulide is not due to interference with FADD-induced apoptosis.

### Knockdown of DR5 decreased TRAIL-and nimesulide-induced caspase-8 activity

**Figure 10.**
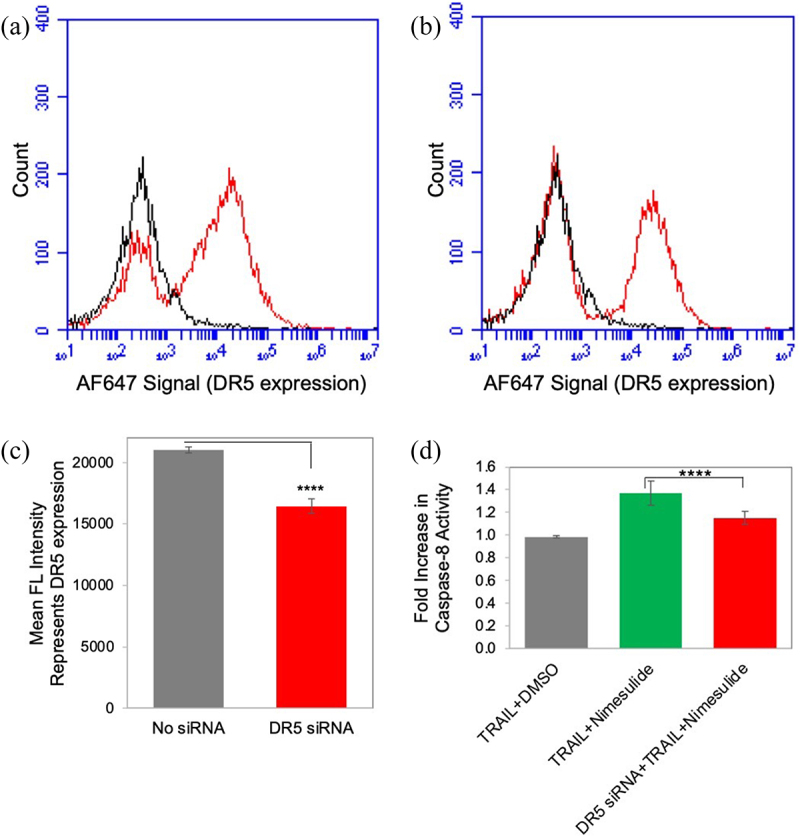
Effect of nimesulide on DR5 siRNA transfected cells. FACS data demonstrate surface expression of DR5 on Panc1 cells (a) and with DR5 siRNA transfected Panc1 (b) cells. (c) Mean FL intensity of transfected and untransfected panc1 cells. Cells were transfected with DR5 siRNA oligonucleotides (25 nM) using lipofectamine 3000. Transfected cells were incubated for 48 hours before further analysis. DR5 expression was determined using anti-DR5 antibody and analyzed by flow cytometry. (d) Caspase-8 activity was measured in siRNA transfected and untransfected panc1 cell. Cells were treated with TRAIL (0.1 μg/ml) +DMSO or TRAIL+nimesulide (50 μM). Data are presented as mean ± standard deviation (N = 3) ****P < .0001 compared to TRAIL nimesulide control by two-tailed unpaired t test.

To determine the specificity of nimesulide in the enhancement of TRAIL-induced apoptosis, we downregulated DR5 expression in pancreatic cancer cells using DR5 siRNA and tested caspase-8 activity in these cells. However, unfortunately, DR5 siRNA knockdown was only partially successful in Panc1 cells (Figure 10a-c). DR5 expression was reduced by 22% after DR5 siRNA transfection of Panc1 cells. We showed that nimesulide-TRAIL-induced caspase-8 activity was decreased in DR5 siRNA transfected cells compared to untransfected cells (Figure 10d). These results suggest that increased caspase-8 activity in the presence of nimesulide originates from DR5 rather than a nonspecific interaction.

## Discussion

Taken together, our results and other published studies suggest that nimesulide has the ability to act on different targets at the same time (e.g., PTEN and DR5).^29–31^ In general, multi-target drugs have attracted attention as potential therapeutic solutions to fight against complex health conditions linked to drug-resistance issues.^61–64^ However, despite their therapeutic promise in cancer, nimesulide and other FDA approved COX-2 inhibitors (e.g., valdecoxib and rofecoxib) have had limited reach (including being banned in several countries) because of their hepatotoxicity and associated cardiovascular problems.^65^ Despite these issues, the European Medicines Agency has confirmed that the hepatic risks associated with nimesulide can be limited by controlling the dose and duration of treatment,^66,67^ and a review of safety standards concluded that nimesulide’s overall benefits outweigh the risks.^22,68^ That said, in part based on these concerns, multiple studies have been done to modify the nimesulide scaffold so as to develop more potent and less hepatoxic anticancer agents.^23,69,70^

As we have now shown, a common feature between nimesulide and DuP-697 is that both compounds promote DR5 clustering and induce apoptosis in TRAIL resistant cancer cells. Exactly how these small molecules achieve this clustering effect remains an important, unanswered question. Despite the intact expression of DR5, pancreatic cancer cells are not capable of initiating DR5 clustering (Figures 6) and caspase-8 activity in the presence of ligand alone (Figures 3c-d and supplementary Figure 5). It is interesting to note that nimesulide treatment alone, while promoting DR5 clustering (Figure 6), wasn’t sufficient to induce caspase-8 activity (Figure 3b). Similar results were reported in TRAIL resistant colon cancer cells treated with DuP-697.^20^ It’s likely that while these small molecules can enhance clustering, concomitant TRAIL binding is necessary to force DR5 into a signaling-competent conformational state. Indeed, we have previously suggested that TRAIL binding causes a conformational change in DR5 that occurs in tandem with clustering, and that specific conformational states of the receptor within clusters are necessary for activation; that is, at the molecular scale, not all clusters are the same.^48,71^

While drugs like nimesulide and DuP-697 show promise, there is nonetheless an ongoing need to develop small molecules that enhance TRAIL sensitivity in cancer cells. There are no existing high-throughput screening (HTS) platforms that enable discovery of small molecules that induce DR5 clustering. For example, neither DuP-697 nor bioymifi were identified through screens based on their direct effect on DR5. In the current and previous studies^35,72^ we have shown that it is possible to monitor a combination of DR5 clustering and conformational changes using a cellular TR-FRET-based DR5 biosensor (Figure 7). We recently showed that this biosensor is HTS-compatible and used it to identify DR5 inhibitors from the Selleck library of small molecules.^73^ The screening platform utilizes a high-precision fluorescence lifetime plate reader^74^ (see Methods) that we have also used recently for small molecule discovery targeting TNFR1.^33,75^ Here, we first identified nimesulide as a promising DR5-targeted compound via a pilot HTS screen of the 1280 compounds in the Sigma Aldrich Library of Pharmacologically Active Compounds (LOPAC) library. In this very limited proof-of-principle screen, we identified nine compounds that increased FRET (Supplementary Figure 6A). These hit compounds have interesting known biological actions. Rutaecarpine is a COX-2 inhibitor, and our study provides the first evidence of its relevance in the DR5 pathway.^76^ Etodolac, gossypol, and bropirimine exhibit an antitumor effect on several different cancer cells. SC-514 and etodolac are anti-inflammatory compounds.^77,78^ A3 hydrochloride is a nonselective casein kinase inhibitor,^79^ Isoguvacine hydrochloride is a γ-aminobutyric acid type A receptor agonist and GR 55562 dihydrobromide is a serotonin receptor antagonist.^80^ Functionality of a random subset of the hit compounds were evaluated using caspase-8 assay. Of five compounds tested, only nimesulide increased caspase-8 activity, and the other compounds either decreased or had no effect on caspase activity (Supplementary Figure 6B) and hence did not warrant additional investigation here.

Our cell-based TR-FRET platform was designed around HEK293 cells (a nonmalignant line) because of their ease of transfection and history of success in drug discovery campaigns using a high-sensitivity fluorescence plate reader technology.^33,81^ Future FRET studies will be done in TRAIL-resistant pancreatic cancer cell lines in order to account for at least two important differences in these cells. First, receptor expression levels impact its function^82–84^ and clustering,^85^ and lines expressing the fluorescently labeled receptors should be engineered to match native levels of DR5 in the plasma membrane of resistant pancreatic cancer cells – though this is a non-trivial undertaking. Second, cancer cells are known to have altered phospholipid and cholesterol content in the plasma membrane^86–88^ and we have shown this can alter DR5 clustering and activity.^32^ Even without these potential improvements, the success we have shown here with nimesulide suggests that the existing platform holds promise for ongoing efforts to study small molecule activators of DR5. One limitation of this new technology is that it does not allow a deconvolution of two related phenomena: an increase in FRET could reflect clustering of DR5 or structural rearrangements in the DR5 backbone. Thus, despite the likelihood of false-positives (e.g., small molecules that increase FRET due to non-activating molecular reorganizations), we are hopeful that given larger libraries this platform will be successful in identifying lead compounds that are more potent and more specific – and thus safer – than nimesulide.

## Supplementary Material

Supplemental Material

## Data Availability

The datasets used or analyzed during the current study are available from the corresponding author on reasonable request.

## Statistical Analysis

Statistical analyses were performed using Prism 8.0 software (GraphPad Software, Inc., CA, USA) and the P-values were calculated using a two-tailed unpaired t test or one-way ANOVA.

*p-value<0.05; **p-value<0.01; ***p-value<0.001 and ****p-value<0.0001.
