# Supplementary figures and images for: Nimesulide, a COX-2 inhibitor, sensitizes pancreatic cancer cells to TRAIL-induced apoptosis by promoting DR5 clustering †
Source: Cancer Biol Ther. 2023 Feb 12;24(1):2176692. doi: 10.1080/15384047.2023.2176692 (PMC9928464; doi:10.1080/15384047.2023.2176692)

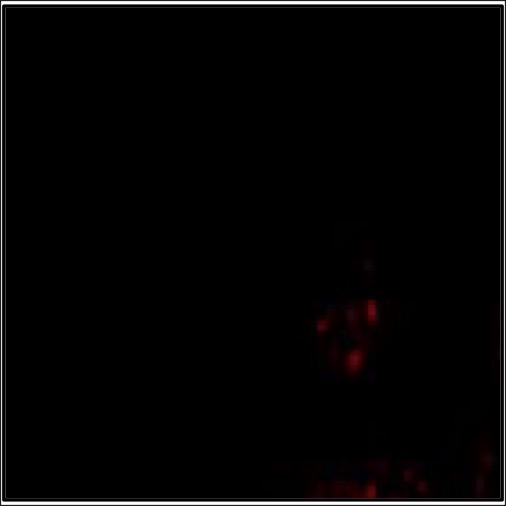

Supplement: Supplemental Material [file KCBT_A_2176692_SM5680.zip › SUPPLEMENTARY_FIGURE_1A.jpg]

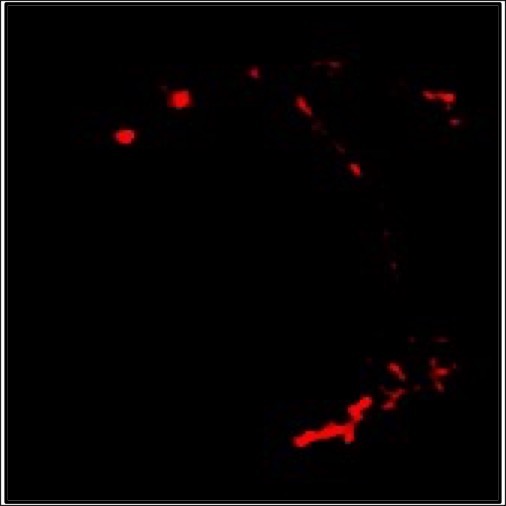

Supplement: Supplemental Material [file KCBT_A_2176692_SM5680.zip › SUPPLEMENTARY_FIGURE_1B.jpg]

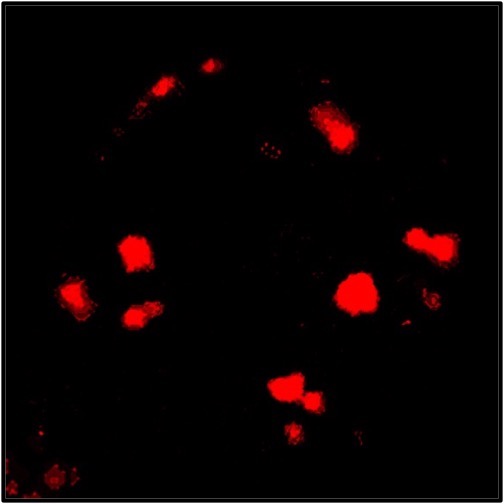

Supplement: Supplemental Material [file KCBT_A_2176692_SM5680.zip › SUPPLEMENTARY_FIGURE_1C.jpg]

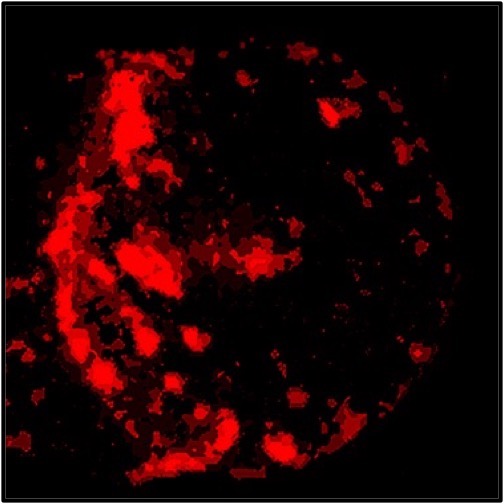

Supplement: Supplemental Material [file KCBT_A_2176692_SM5680.zip › SUPPLEMENTARY_FIGURE_1D.jpg]

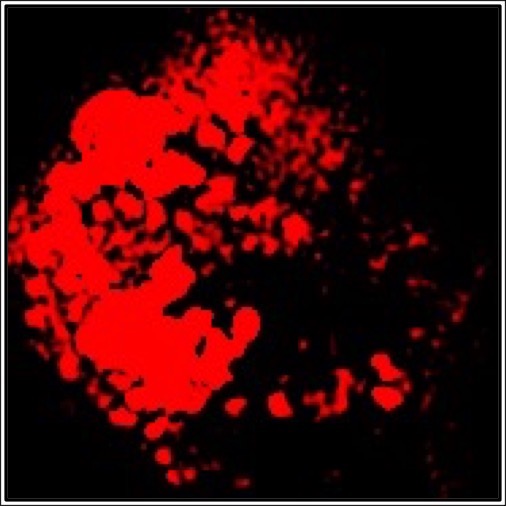

Supplement: Supplemental Material [file KCBT_A_2176692_SM5680.zip › SUPPLEMENTARY_FIGURE_1E.jpg]

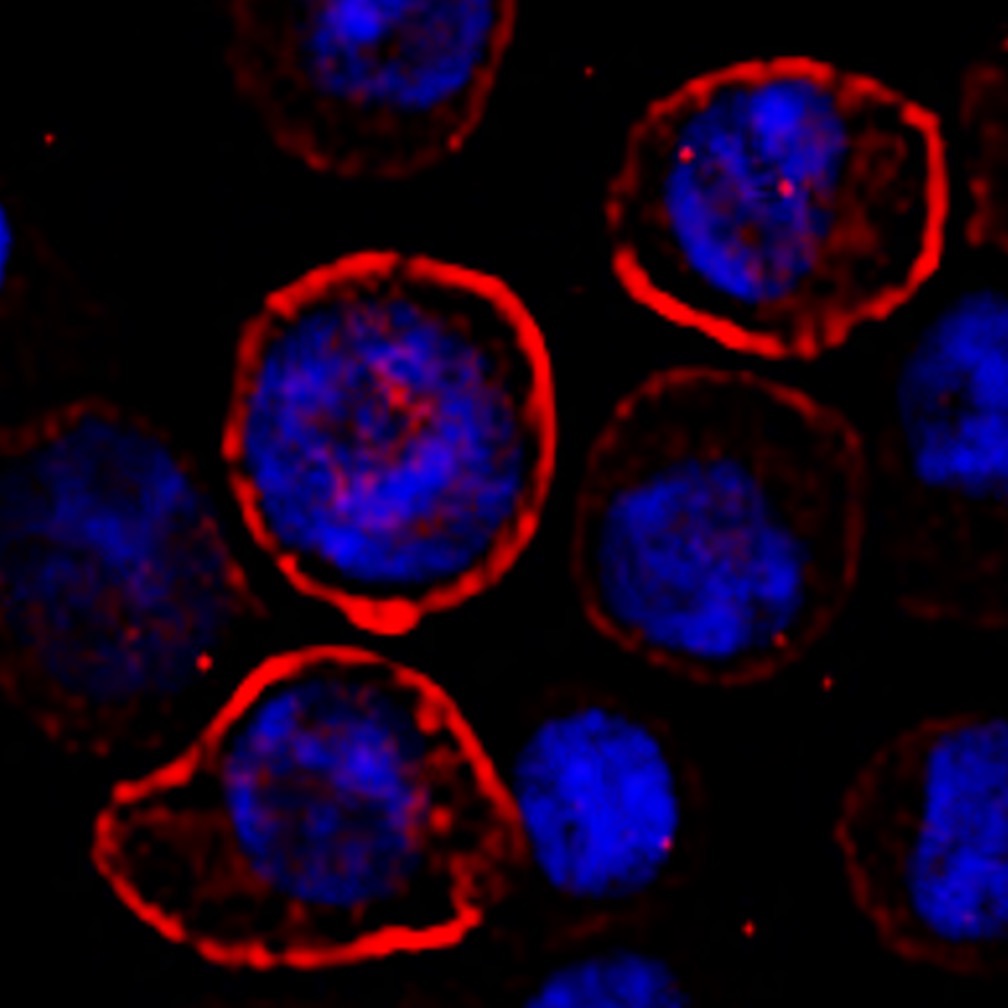

Supplement: Supplemental Material [file KCBT_A_2176692_SM5680.zip › SUPPLEMENTARY_FIGURE_2A1.jpg]

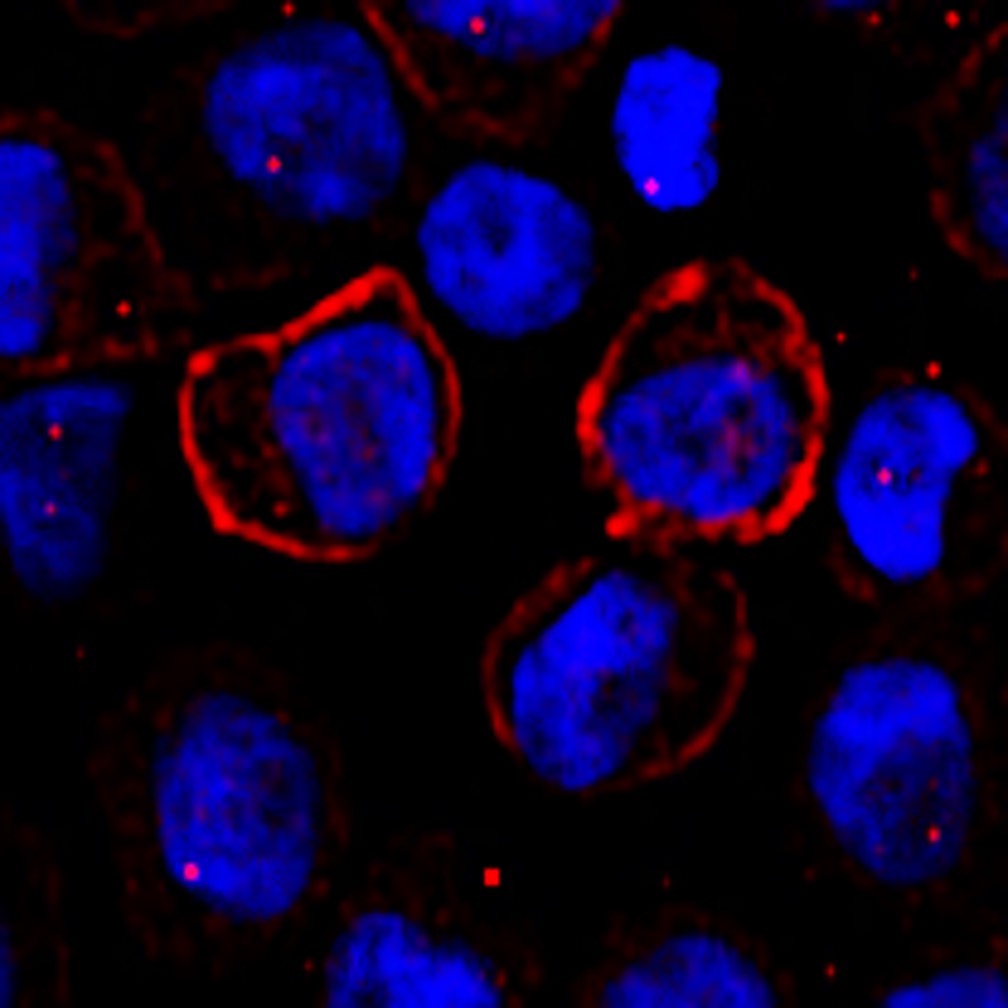

Supplement: Supplemental Material [file KCBT_A_2176692_SM5680.zip › SUPPLEMENTARY_FIGURE_2A2.jpg]

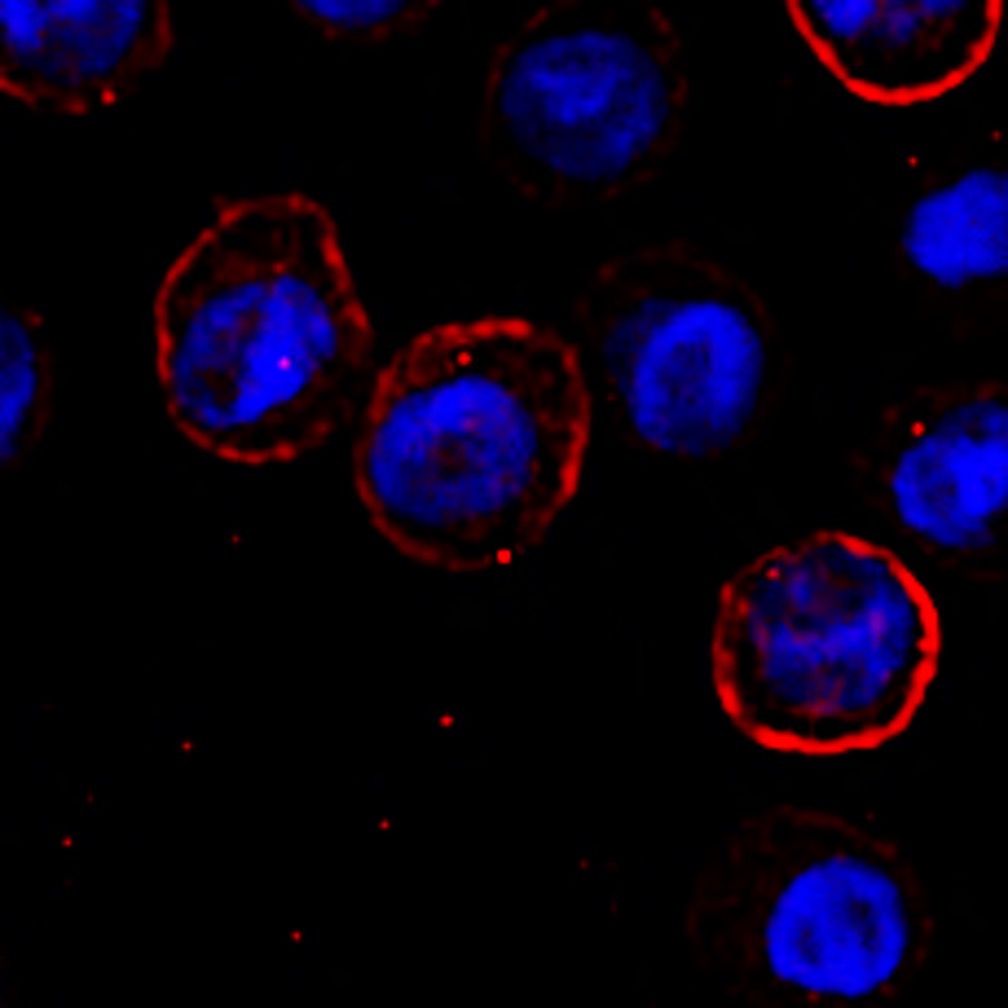

Supplement: Supplemental Material [file KCBT_A_2176692_SM5680.zip › SUPPLEMENTARY_FIGURE_2A3.jpg]

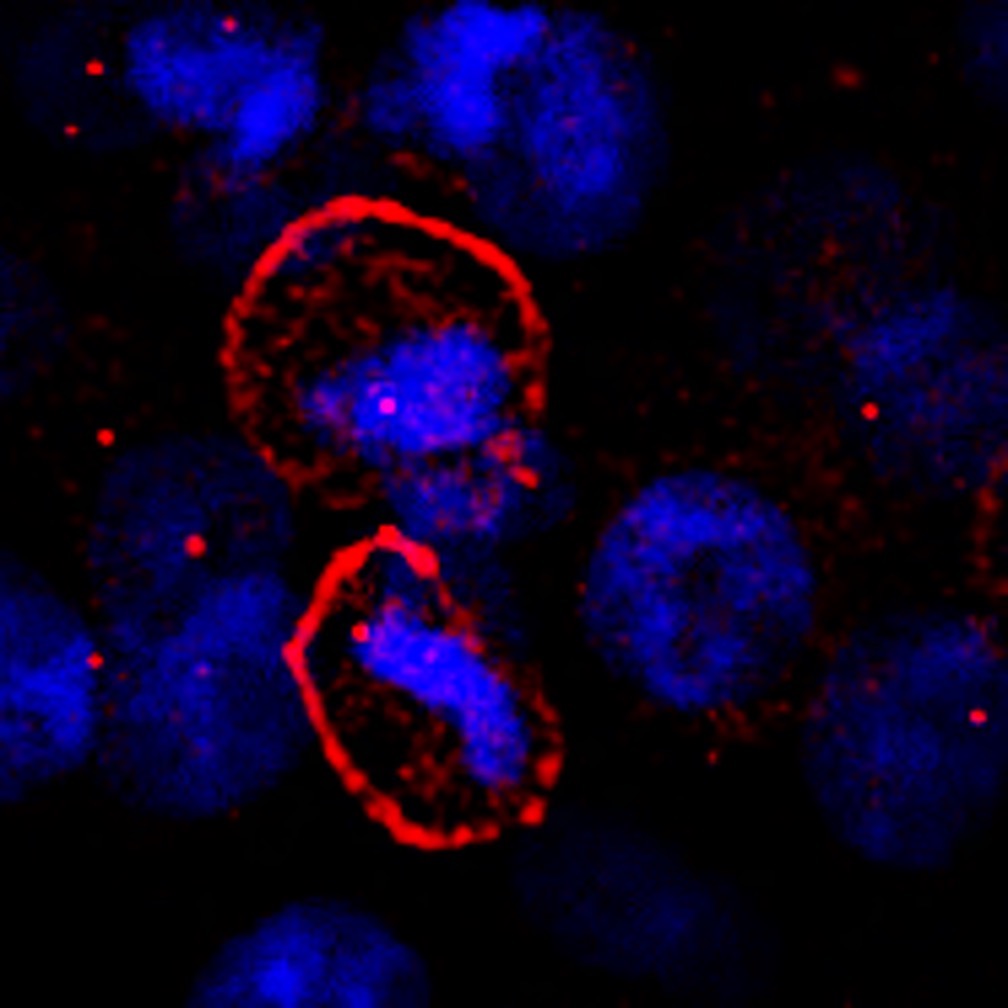

Supplement: Supplemental Material [file KCBT_A_2176692_SM5680.zip › SUPPLEMENTARY_FIGURE_2A4.jpg]

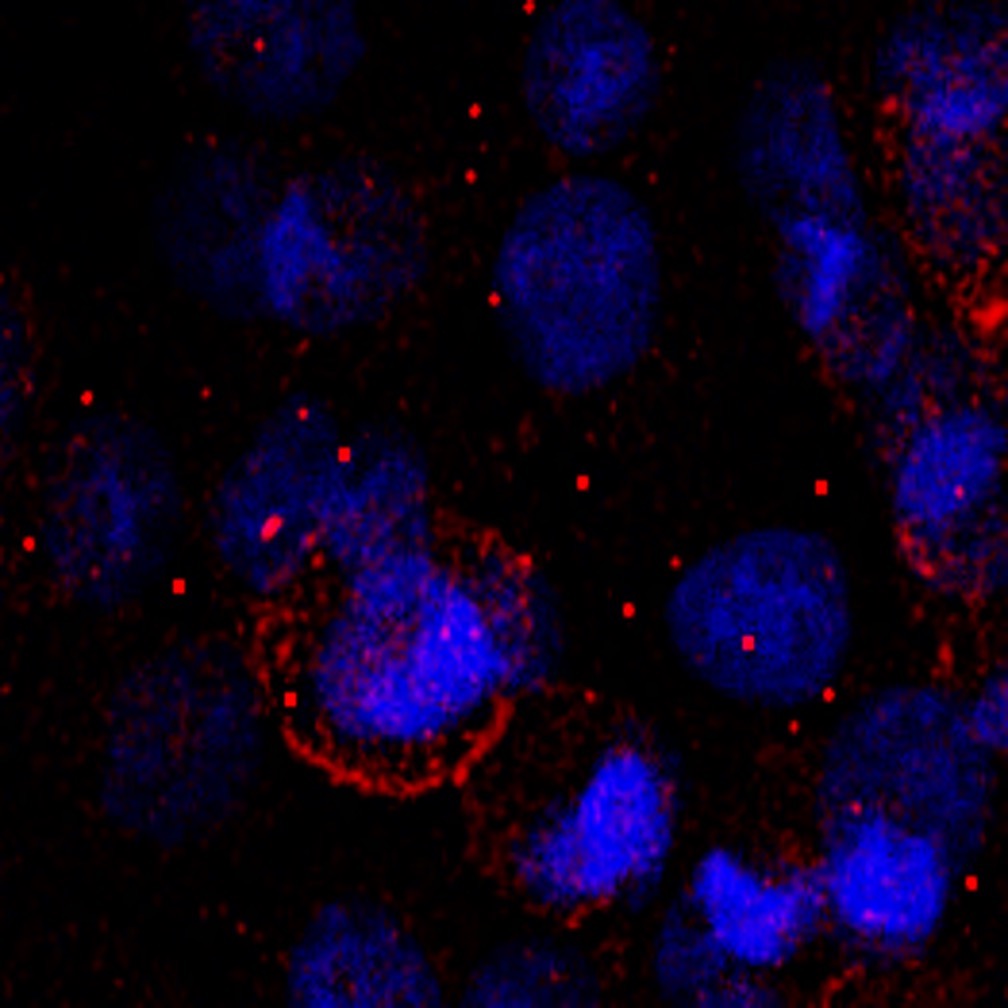

Supplement: Supplemental Material [file KCBT_A_2176692_SM5680.zip › SUPPLEMENTARY_FIGURE_2B1.jpg]

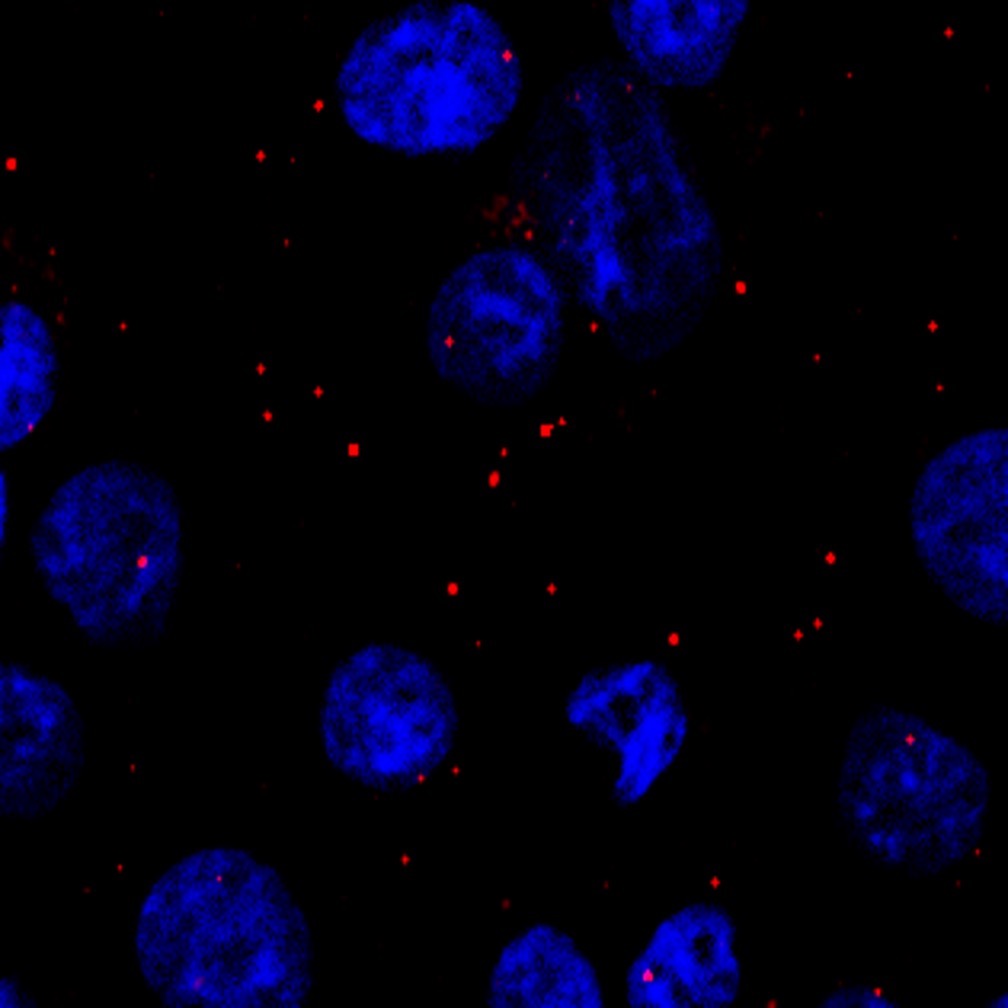

Supplement: Supplemental Material [file KCBT_A_2176692_SM5680.zip › SUPPLEMENTARY_FIGURE_2B2.jpg]

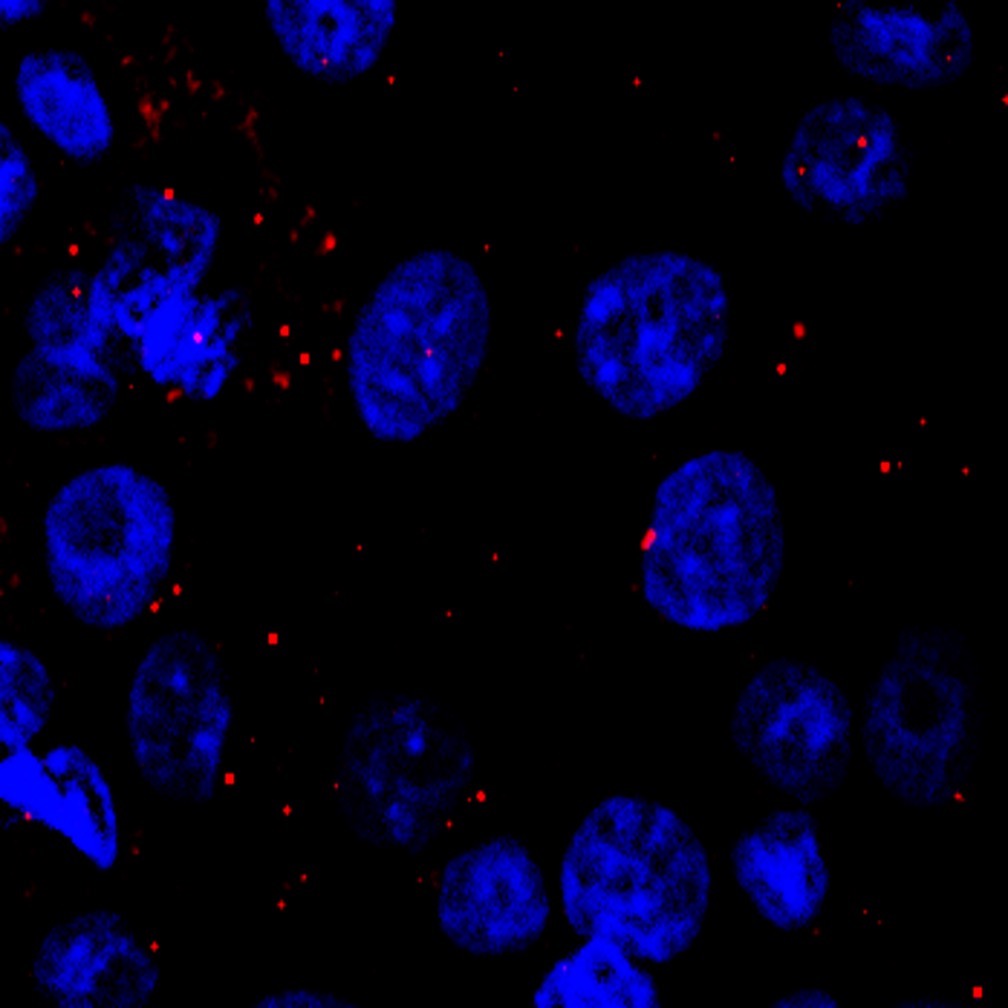

Supplement: Supplemental Material [file KCBT_A_2176692_SM5680.zip › SUPPLEMENTARY_FIGURE_2B3.jpg]

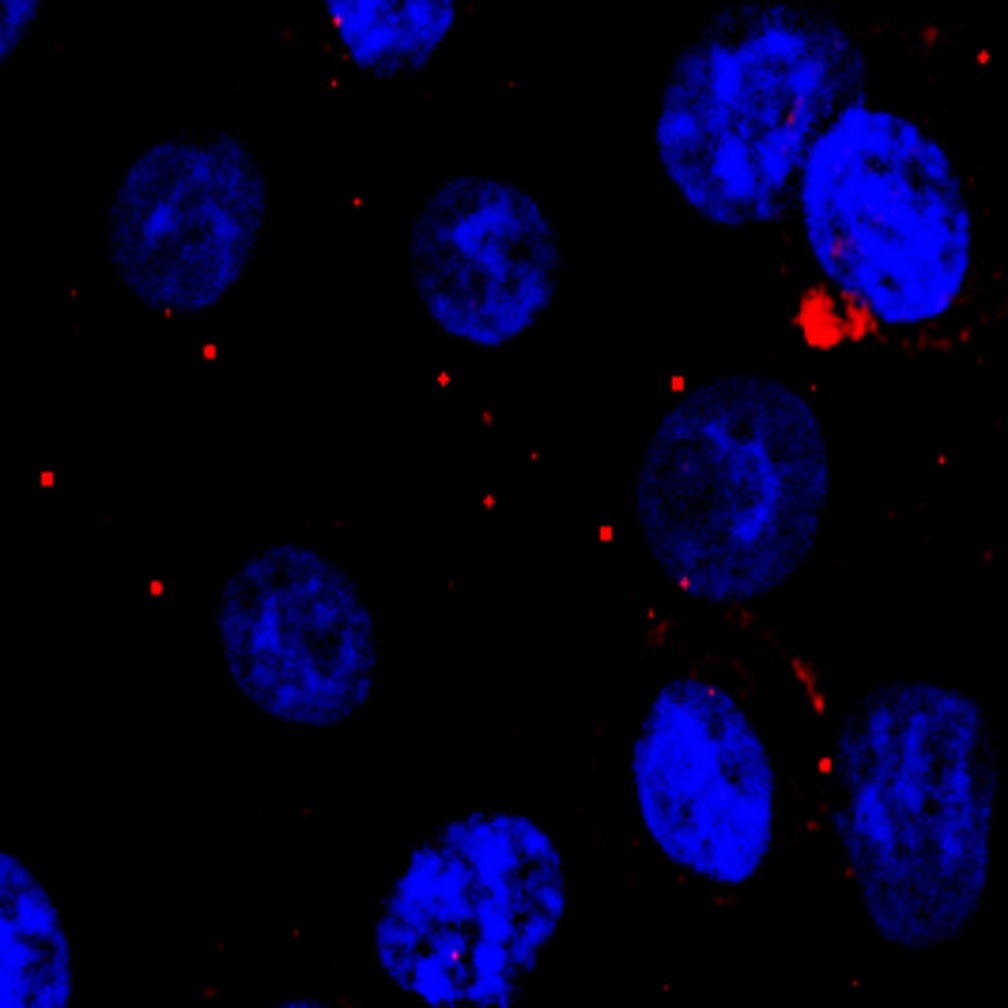

Supplement: Supplemental Material [file KCBT_A_2176692_SM5680.zip › SUPPLEMENTARY_FIGURE_2B4.jpg]

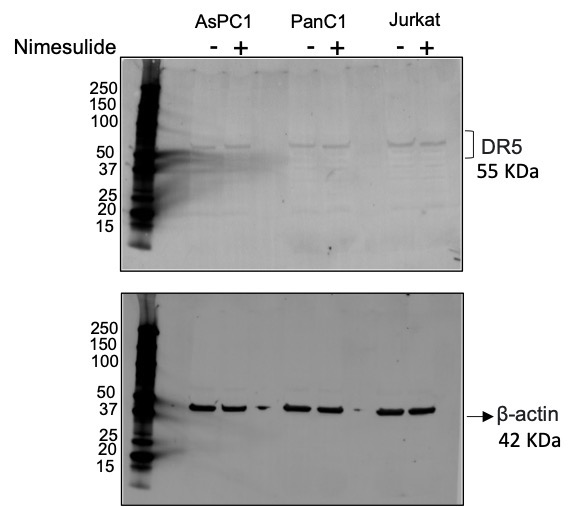

Supplement: Supplemental Material [file KCBT_A_2176692_SM5680.zip › SUPPLEMENTARY_FIGURE_3A.jpg]

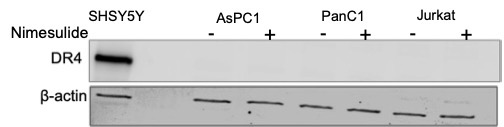

Supplement: Supplemental Material [file KCBT_A_2176692_SM5680.zip › SUPPLEMENTARY_FIGURE_3B.jpg]

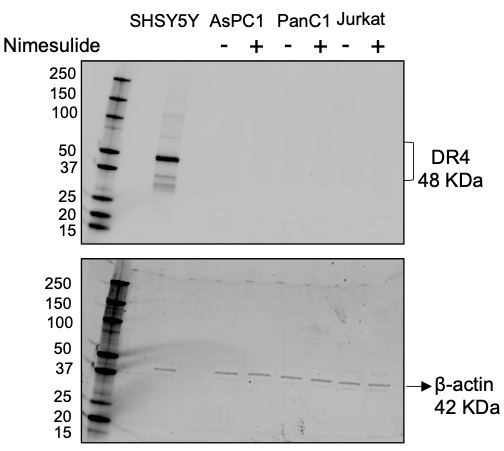

Supplement: Supplemental Material [file KCBT_A_2176692_SM5680.zip › SUPPLEMENTARY_FIGURE_3C.jpg]

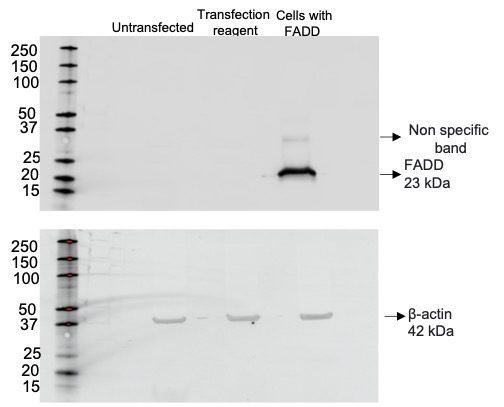

Supplement: Supplemental Material [file KCBT_A_2176692_SM5680.zip › SUPPLEMENTARY_FIGURE_4.jpg]

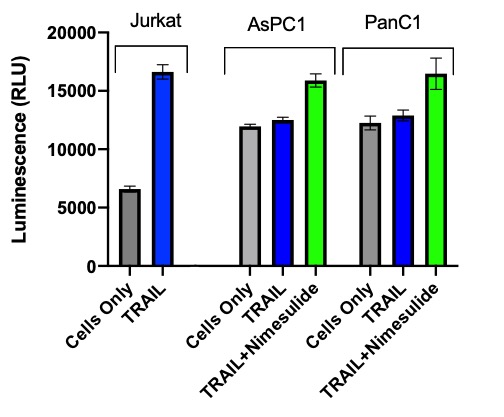

Supplement: Supplemental Material [file KCBT_A_2176692_SM5680.zip › SUPPLEMENTARY_FIGURE_5.jpg]

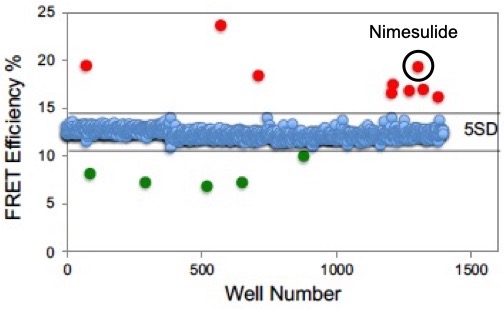

Supplement: Supplemental Material [file KCBT_A_2176692_SM5680.zip › SUPPLEMENTARY_FIGURE_6A.jpg]

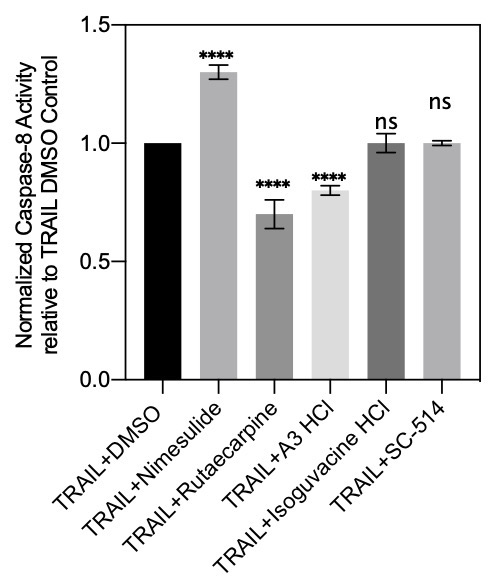

Supplement: Supplemental Material [file KCBT_A_2176692_SM5680.zip › SUPPLEMENTARY_FIGURE_6B.jpg]
